# Targeting of Glycosaminoglycans in Genetic and Inflammatory Airway Disease

**DOI:** 10.3390/ijms23126400

**Published:** 2022-06-08

**Authors:** Robin Caird, Michael Williamson, Azeez Yusuf, Debananda Gogoi, Michelle Casey, Noel G. McElvaney, Emer P. Reeves

**Affiliations:** Irish Centre for Genetic Lung Disease, Department of Medicine, RCSI University of Medicine and Health Sciences, Beaumont Hospital, D09 YD60 Dublin, Ireland; robincaird20@rcsi.ie (R.C.); michael.williamson@cuh.ie (M.W.); debanandagogoi@rcsi.ie (D.G.); michellecasey@rcsi.ie (M.C.); gmcelvaney@rcsi.ie (N.G.M.); emerreeves@rcsi.ie (E.P.R.)

**Keywords:** glycosaminoglycans, cystic fibrosis, chronic obstructive pulmonary disease, COVID-19, asthma, inflammation

## Abstract

In the lung, glycosaminoglycans (GAGs) are dispersed in the extracellular matrix (ECM) occupying the interstitial space between the capillary endothelium and the alveolar epithelium, in the sub-epithelial tissue and in airway secretions. In addition to playing key structural roles, GAGs contribute to a number of physiologic processes ranging from cell differentiation, cell adhesion and wound healing. Cytokine and chemokine–GAG interactions are also involved in presentation of inflammatory molecules to respective receptors leading to immune cell migration and airway infiltration. More recently, pathophysiological roles of GAGs have been described. This review aims to discuss the biological roles and molecular interactions of GAGs, and their impact in the pathology of chronic airway diseases, such as cystic fibrosis and chronic obstructive pulmonary disease. Moreover, the role of GAGs in respiratory disease has been heightened by the current COVID-19 pandemic. This review underlines the essential need for continued research aimed at exploring the contribution of GAGs in the development of inflammation, to provide a better understanding of their biological impact, as well as leads in the development of new therapeutic agents.

## 1. Introduction

Inflammation is part of the body’s natural defence system employed to eradicate harmful entities that may cause injury [1]. Inflammation in its acute form is used to recruit immune cells to facilitate wound healing. However, as the airways are constantly exposed and bombarded with different inhaled stimuli such as particulate matter from the atmosphere and pathogens, chronic inflammation may arise. Under these circumstances, tissue injury evokes continuous recruitment of immune cells that induce sustained secretion of inflammatory molecules that can cause further damage to the airways [2], giving rise to a range of chronic respiratory diseases. Chronic respiratory diseases are a major public health concern globally, accounting for 4.7% of the global disability and 7% of all deaths worldwide [3,4].

The extracellular matrix (ECM) in the airways is a three-dimensional scaffold that provides physical support for cells as well as biochemical cues for cellular processes such as cellular morphogenesis/differentiation and tissue homeostasis [5]. The ECM is comprised of macromolecules, mainly structural proteins and glycosaminoglycans (GAGs), which are polysaccharides made up of repeating disaccharide units of galactosamine, *N*-acetylgalactosamine-4-sulphate, galactose or galacturonic acid. Major types of GAGs include heparin/heparin sulfate, chondroitin sulphate, dermatan sulphate, hyaluronic acid and keratin sulphate. In the airways and the lungs, GAGs are usually linked covalently with core proteins known as proteoglycans, such as syndecan (chondroitin and heparin sulfate), decorin (dermatan sulfate) and glypican (heparan sulfate). Furthermore, GAGs also interact with other proteins within the ECM including chemokines, cytokines, and adhesion molecules [6]. Due to their usual association with the cell membrane, GAGs act as cell surface receptors or co-receptors to capture ligands required for activating downstream signalling. For example, heparan sulfate (HS) and its proteoglycan syndecan act for molecular capture of fibroblast growth factor (FGF) receptor (FGFR) to facilitate its internalisation and endosomal sorting in an FGF-dependent manner [7]. Cell surface GAGs also bind to chemokines that are released consequent to tissue injury to direct leukocyte migration and promote inflammation that may influence tissue repair or healing [8]. These types of inflammatory response are a hallmark of various respiratory diseases. Over the past two decades, increasing pathophysiological roles of GAGs have been uncovered. This review aims to discuss recent developments in the role of GAGs in respiratory diseases as well as recent advances in utilisation of GAGs as therapeutic targets. A review of the literature was conducted using the PUBMED database, Google Scholar and The Cochrane Library databases.

## 2. Glycosaminoglycans in the Airways: Physical Properties

To facilitate gas exchange the lung architecture possesses certain physical attributes. Surface area dictates the amount of gas exchanged and the lungs therefore have the largest epithelial surface in the body. In adults, the lungs together contain approximately 2400 km of airways and 300–500 million alveoli; with a total surface area of approximately 70 m^2^ [9]. The lungs are highly dynamic and require the ability to inflate and deflate quickly without being damaged or losing structural integrity, however, the wall of each alveolus must also be extremely thin to allow diffusion to occur with maximum efficiency. The composition of this unique tissue is therefore of great importance.

**Figure 1:**
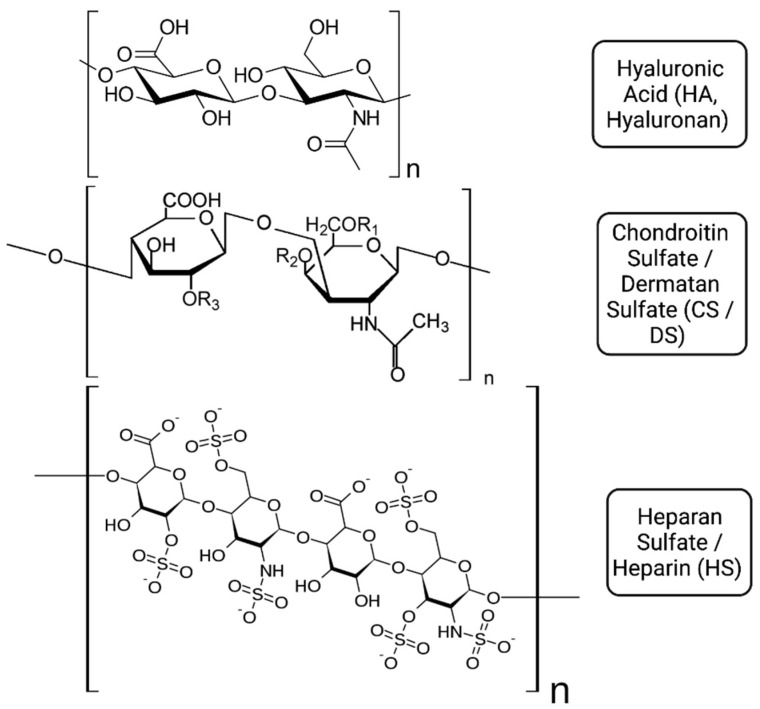
Repeating subunits of hyaluronic acid/hyaluronan, chondroitin sulfate/dermatan sulfate and heparan sulfate/heparin.

The alveolar wall is composed of an epithelial cell layer, the capillary basement membrane and endothelial cells, and the ECM, which is the space between the capillary and alveolar epithelia. The ECM is a framework of macromolecules such as collagen fibres, elastic fibres and GAGs [10]. GAGs, a major component of the ECM, are long linear polysaccharides made up of a repeating disaccharide unit. The repeating unit, which can range in length from 1 to 25,000 disaccharide units, consists of a hexose or a hexuronic acid, linked to a hexosamine, forming galactose, galactosamine, *N*-acetylgalactosamine-4-sulphate or galacturonic acid. GAGs fall into four categories based on the repeating disaccharide [11]. Hyaluronic Acid (HA, Hyaluronan) is composed of repeating units of d-glucuronic acid-(*β*1-3)-*N*-acetyl-d-glucosamine; Chondroitin Sulfate/Dermatan Sulfate (CS/DS) with repeating units of d-glucuronic acid-(*β*1-3)-*N*-acetyl-d-galactosamine; Heparan Sulfate/Heparin (HS) with repeating units of d-glucuronic acid-(*β*1-4)-*N*-acetyl-d-glucosamine; and Keratan Sulphate (KS) with repeating units of d-galactose-(*β*1-4)-*N*-acetyl-d-glucosamine (Figure 1). GAGs are ubiquitously expressed and all four groups are represented in the human lung.

GAGs have been implicated in the pathology of many diseases, including neurodegenerative diseases such as amyloid lateral sclerosis (ALS) and Alzheimer’s, cardiovascular diseases, different types of cancer, some pulmonary diseases and cystic fibrosis (CF) [12,13,14,15,16]. GAGs are of particular concern in CF as they are instrumental to the recurrent respiratory infections that contribute to morbidity and mortality [17]. The most common GAG is HS, accounting for 40–60% of all GAGs. Of the remaining groups CS/DS accounts for 31%, HA 14%, and heparin 5% [18]. HS, KS and CS/DS are termed sulphated GAGs, whereas HA is the only non-sulphated GAG. The sulphated GAGs are formed intracellularly within the Golgi apparatus where they are elongated by adding anywhere from 20–200 disaccharide units. They are modified at this stage to provide wide variety within subgroups, for example, in degrees of sulfation and acetylation [19]. This is highly varied in HS, and in comparison, less variation occurs within the KS and CS/DS classes. Once formed, the majority of sulphated GAGs are bound to protein cores forming proteoglycans, although HS, which is found in nearly every cell in the human lung, can also be secreted in an unbound form as a soluble GAG [20]. In this regard, HS is found on cell surfaces as HS proteoglycans (HSPG), which is formed via the linear covalent bonding of HS molecules to a core protein [21]. However, HA is unique amongst the GAGs as it is not formed in the Golgi apparatus but in the cell membrane. Moreover, HA is the only GAG that is not sulphated and is larger than the other GAGs containing up to 10,000 residues compared to 20–200 residues of the other GAGs. Unlike the other GAGs that are tightly associated with core proteins of proteoglycans, HA is found as long, viscous, tangled chains [22].

All GAGs are highly anionic with multiple charges along the chains, a property that makes them a vital part of the connective tissue matrix. Their viscosity and multiple anionic charges allow them to structurally stabilize the thin alveolar walls, resisting the constantly changing forces caused by respiration [23]. HS, in particular, is a key component of the ECM, which binds laminin to support the basement membrane structure [24]. Due to their highly charged nature, GAGs are able to regulate ECM hydration, osmotic pressure and flow resistance by attracting and binding to water molecules [25]. They ensure structural stability within the lung by providing protection against enzymatic breakdown and encouraging lung tissue repair following acute injury, by actively regulating the fibro-proliferative response which requires the enzymes HA synthase 2 and hyaluronidase 2 [26,27]. Furthermore, within the pulmonary alveolus, HA plays a crucial role in the stabilisation of the alveolar surface structure by interacting with surfactant mainly phospholipids [28].

## 3. Biological Roles and Protein Interactions of Glycosaminoglycans

### 3.1. GAGs and Pro-Inflammatory Protein Interactions

GAGs play a major role in the control of chemokine-mediated inflammation, which may impact mechanisms of inflammation in different diseases of the airways. GAGs strongly bind chemokines in an interaction that is crucial for their biological effects. For instance, plasma membrane CXCR1 is a G-protein coupled receptor that binds IL-8 to mediate neutrophil recruitment from the vascular system [29], and migration to sites of inflammation [30]. Early investigations employing in vitro models utilised transendothelial gradients of soluble chemokines to stimulate neutrophil migration, and the in vivo process was initially assumed to be the same [31]. However, neutrophils are recruited from the blood, and their ability to extravasate from the arterial blood stream, makes it highly unlikely that soluble chemokines could remain in place for sufficient duration to act as an ongoing trigger. Instead, chemokines immobilized on the luminal endothelial cell surface, and available in solid phase to circulating neutrophils, provide the necessary gradient for a sustained neutrophilic response. The majority of cell types that produce chemokines are extravascular; therefore, chemokines must transfer to the luminal surface before presentation to leukocytes. Electron microscopy studies have revealed that IL-8 is internalized in venous endothelial cells and transcytosed to the luminal surface to be fixed on the tips of endothelial cell villi, where it can come into direct contact with neutrophils [32]. GAGs are the main structures that immobilise chemokines allowing an immobilised gradient from vascular endothelium to airway epithelium [33,34].

IL-8 bound to GAGs on the vascular endothelial surface rapidly increases integrin adhesiveness, followed by firm adherence of immune cells to the endothelium [35,36]. Subsequently, a gradient of chemokine signalling encourages migration from the vascular endothelium toward the inflamed tissue [37]. HS, the most abundant GAG in the lungs, also has the highest affinity for binding to IL-8, and in a murine study, mice deficient in endothelial HS displayed impaired neutrophil infiltration [38]. These studies confirm the distinct roles of endothelial HS, including binding and presentation of HS-associated chemokines at the luminal endothelium, HS’s role as an L-selectin ligand during neutrophil rolling, and facilitation of chemokine transcytosis [39]. GAGs themselves do not have chemotactic properties, however, binding to HS or heparin significantly enhances neutrophil responses to IL-8 [40]. This is in part due to the ability of the normally monomeric IL-8 to dimerise when bound to GAGs. Dimerization substantially increases the concentration of IL-8 bound in lung tissue creating a powerful stimulus to neutrophil migration [41,42]. In chronic airway diseases such as COPD, CF and non-CF bronchiectasis, a consequence of increased neutrophil influx is the release of serine proteases such as neutrophil elastase, which is an important biomarker of disease progression in CF [43,44]. NE exacerbates CF inflammation by upregulating secretion of pro-inflammatory chemokines such as IL-1α, IL-1β, IL-8, IL-33 and TNF-α, resulting in further neutrophil chemotaxis, in a cycle of inflammation [44,45,46].

The expression and breakdown of GAGs are dynamic processes that can induce a tissue specific inflammatory response. Kuschert and Colleagues (1999) studied GAG expression on human umbilical vein epithelial cells (HUVECs) and their interaction with the chemokines RANTES, MCP-1, IL-8 and MIP-1R [47]. It was found that GAGs bound to chemokines with different affinities, as varying ionic strength was required to cause their dissociation from heparin−sepharose. Furthermore, the chemokines showed preferential binding to different GAGs. RANTES for example was more likely to bind to CS/DS compared to IL-8. The described preferential interactions may reflect differential expression of GAGs in different tissue and may also be responsible for the difference in immune cell response. Finally, biological activities of chemokines may be altered depending on the types of GAGs they bind to. While binding to immobilised GAGs does not alter the biological activities of IL-8, binding to soluble GAGs inhibits IL-8 interaction with CXCR1, CXCR2, and CCR1 [47], and similarly, soluble heparin inhibits CXCL10 induced T-cell chemotaxis [48].

### 3.2. GAG Proteases Interactions

In airway diseases such as CF and COPD, poorly controlled inflammation can result in a severe protease–antiprotease imbalance, largely due to the increased activities of NE. NE is a potent enzyme capable of degrading components of connective tissue, including elastin [49,50]. HS and heparin, and to a lesser extent HA and CS, may provide protection to the structural integrity of the lungs by strongly inhibiting leukocyte elastase activity and preventing breakdown of elastin in the lung [51]. The exposure of HS to elastase results in HS fragments due to partial degradation of the core protein [52]. Similarly, as with HA fragments, HS fragments are found in areas of lung damage and inhibit NE by a tight-binding, competitive mechanism. This effect is length dependent and a minimum fragment length of 12–14 saccharides is needed for inhibition, and inhibition increases with increased chain length [53]. Of interest, nebulised heparin has long been recognized as having anti-inflammatory effects that are beneficial in asthma [54]. Semi-synthetic low-molecular-weight heparin inhibits both human NE and cathepsin G by binding to these enzymes. This inhibition again, is dependent on heparin length and sulfation, suggesting that synthesised ‘best fit’ GAGs may be regulators of airway inflammation [55]. The positive effects of GAGs on protease–anti-protease balance is not restricted to NE binding. Secretory leukocyte peptidase inhibitor (SLPI), one of the main protective anti-proteases active at the airway epithelium, is dysfunctional due to oxidization by *N*-chlorotaurine, an oxidant generated by activated neutrophils. However, in the presence of an iduronate-containing glycosaminoglycan, such as heparin, HS, or DS, this impairment is substantially reversed. Analysis revealed that GAGs increased the interaction of oxidized SLPI and NE and also acted to stabilise the enzyme-inhibitor complex [56].

## 4. The Role of Glycosaminoglycans in Airways Disease

### 4.1. An Introduction to Cystic Fibrosis

CF is a genetic disease caused by mutations in the CF transmembrane conductance regulator gene (CFTR), which is inherited in an autosomal recessive manner. As such there is a 25% chance that a child of parents that are heterozygous for CF will develop CF. CF affects approximately 70,000 individuals worldwide, most commonly in those of North European descent [57]. The clinical signs of CF can manifest in multiple organs, including the lungs, sinuses, sweat glands, liver, pancreas, and digestive tract [57].

**Figure 2:**
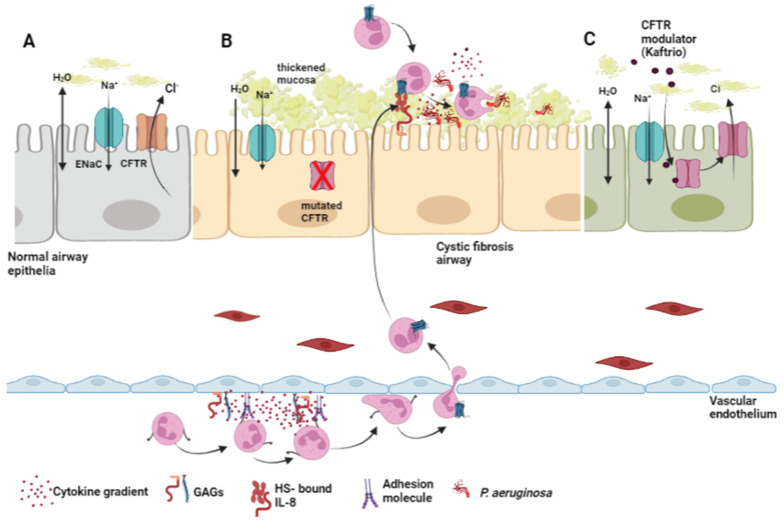
CF airways disease involves cytokine gradients created by glycosaminoglycans (GAG) which facilitate neutrophil extravasation in cystic fibrosis. (**A**) In healthy airway epithelial cells, fully functional CFTR plays a key role in regulating hydration of the airway surface liquid (ASL) layer. CFTR ion channels move chloride ions (Cl^−^) from inside the cell to outside the cell, which attracts a layer of water, facilitating cilia movement and allowing passage of mucus up and out of the airways. (**B**) In patients with CF, due to a lack of CFTR function, Cl^−^ secretion is impaired and Na^+^ absorption through ENaC is upregulated resulting in dehydration of the ASL, thick mucus accumulating and bacterial colonization. Mucus viscosity is further increased by GAGs, which are increased in the CF airways and are also important for regulation of cytokine gradients. Neutrophils extravasate through endothelial cells of the blood vessels using cytokine gradients created by GAGs including chondroitin sulfate, heparin sulfate and hyaluronic acid. GAG-bound IL-8 facilitates the recruitment of neutrophils through binding of IL-8 to CXCR1. Recruited neutrophils fail to kill invading bacteria, and sustained recruitment leads to neutrophil dominated inflammation. (**C**) CF care includes small molecule drugs targeting the underlying CFTR protein defect, such as the CFTR modulator therapy Trikafta (Elexacaftor/Tezacaftor/Ivacaftor combination, Vertex Pharmaceuticals, also known as Kaftrio), which demonstrates significant improvement in lung function.

The main morbidity and mortality in CF are associated with lung disease, within the airways, apical membrane CFTR defect results in increased water absorption from the airway lumen [58], reduced periciliary layer (PCL) volume and increased hyper-viscosity of mucosal secretions. Concentration of mucus and a reduction in the height of the PCL results in compressed cilia and reduced mucolytic clearance giving rise to mucus accumulation and airway plugging. This thick mucus layer supports chronic bacterial colonisation, most notably *Pseudomonas aeruginosa* (*P. aeruginosa*) (Figure 2). This results in increased inflammation, progressive tissue damage and decline in lung function.

The current treatment modalities for the symptoms of CF include antibiotics, mucolytics, anti-inflammatory agents and lung transplantation. Non-pharmacological interventions include airway clearance techniques, chest physiotherapy and high-energy diet [59]. The most significant advance in the treatment of CF in recent years has been the emergence of CFTR modulators including CFTR potentiators [60,61,62] and CFTR correctors [63]. These targeted therapies have had an enormous impact on both quality of life and life expectancy for patients with CF.

#### 4.1.1. The Role of Glycosaminoglycans in the Pathology of Cystic Fibrosis

Although abundant throughout the body, GAG expression is elevated in CF [64]. HS is significantly more abundant in epithelial and endothelial basement membranes of patients with CF, even more so when compared to COPD [65]. The HS found in CF is also more likely to be intact and provides vast opportunity for the retention and stability of IL-8 and sustained neutrophil recruitment to the airways (Figure 2) [65]. Serum HA concentrations are significantly increased in patients with CF and often observed in CF-associated liver disease, with higher concentrations correlating with more severe liver damage [66]. Abnormal CFTR function results not only in increased expression of GAGs but also in atypical degrees of sulphation. This was noted in airway secretions [67], and respiratory epithelium of patients with CF [68].

The enzyme arylsulfatase B (ASB) catalyses hydrolysis of the sulfate ester of *N*-acetylgalactosamine 4-sulphate from DS/CS. In patients with CF, there is decreased ASB activity, which may partly explain the over-sulphation of GAGs [69]. ASB activity was measured in CF cell lines and following correction of the CFTR defect, with 40% increase in ASB activities recorded in the latter [69]. Moreover, IL-8 increases in bronchial epithelial cells after ASB silencing, indicating that ASB activity may impact IL-8–mediated inflammatory responses [70]. A decline in ASB enzyme activity by approximately 75% was observed coupled with an increase in the cellular CS content and cell-bound IL-8 protein [70]. Collectively, these findings highlight a potential role for GAG sulphation in regulation of cytokine secretion in the CF lung. Additional studies have since demonstrated that GAGs play an important role in regulating the inflammatory response, and targeting of GAGs can reduce levels of IL-8 in CF airway samples [64,71]. Of importance, the impact of CFTR modulators on the abundance or function of GAGs is poorly understood. An in vitro study by Bhattacharyya and colleagues investigated the potential of CFTR modulators to reduce the accumulation of CS in CF through the upregulation of ASB [72]. It was found that ASB activity was elevated in CF cells treated with VRT-532 (an experimental CFTR potentiator), and that IL-6 activity and CS accumulation decreased post-treatment [72].

#### 4.1.2. Glycosaminoglycans and the Management of Cystic Fibrosis

As discussed, GAGs are involved in regulating inflammation via binding to pro-inflammatory mediators, interacting with proteolytic enzymes and directly influencing cell signalling. In CF, GAG expression and sulphation is known to be abnormal which contributes to a poorly regulated inflammatory profile through their influence on chemokine activities. This degree of involvement in CF lung disease has made GAGs attractive as a possible means of therapeutic intervention. Specific GAG mimics have been manufactured to interact with chemokines [73], growth factors [74], proteases [75], and adhesion molecules [76]. Several synthetic GAGs have been tested in recent years for treatment of CF-related phenotypes. Low-molecular-weight polysulphated HA has been shown to block LPS-stimulated release of cytokines such as IL-6, IL-12, TNFα, and MCP-1 in macrophage, and increase expression of superoxide dismutases 2 and 3 [77]. A sulphated semisynthetic low-molecular-weight HA, GM-1111, was tested for anti-inflammatory potential in a mouse model of rhinosinusitis. GM-1111 was found to block mucosal infiltration of neutrophils and mast cells, significantly reducing epithelial apoptosis [78]. More specific to CF, in mice carrying the *F508del* CFTR mutation, nebulized HA significantly reduced protein levels of the pro-inflammatory mediators TNFα, MIP-2, and MPO in lung tissue samples [79], thus demonstrating a direct link between HA and control of lung inflammation in CF. Nebulisation of hypertonic saline (HTS) has also been used to disrupt the interaction between IL-8 and GAGs, thereby assisting resolution of lung inflammation in patients with CF [64]. Finally, nebulized HA was also assessed for its ability to aid in the delivery of other treatments, particularly HTS. Addition of HA to HTS for nebulization improved patient perceptions of “pleasantness” and overall tolerability of nebulized HTS in patients with CF. Importantly, it lessened the bronchoconstriction associated with HTS and reduced the need for β-adrenergic bronchodilators [80,81,82,83].

### 4.2. Glycosaminoglycans in Chronic Obstructive Pulmonary Disease

**Figure 3:**
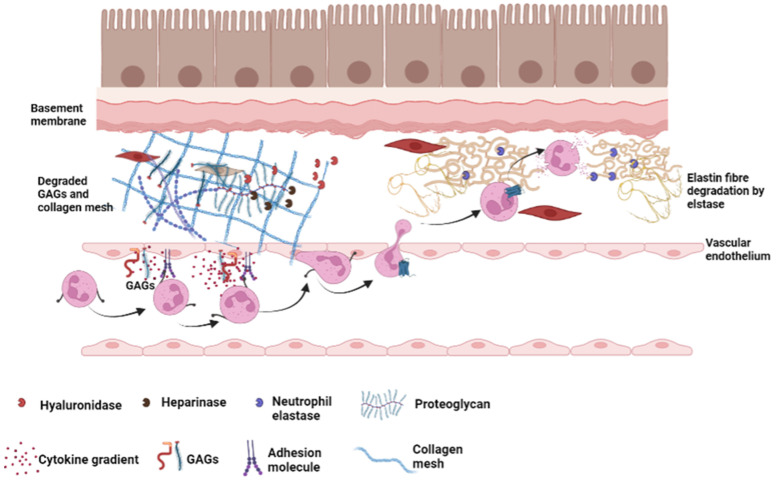
Remodelling of the extracellular matrix in COPD: Neutrophils that extravasate through the endothelial cells to the airways are drawn by cytokine gradients created by glycosaminoglycans (GAGs) on the endothelial surface. The neutrophils release neutrophil elastase (NE) that degrades elastin fibres in the ECM. Within the ECM, there is high activity of GAG lyases such as hyaluronidase and heparinase that cause degradation of GAGs leading to structural changes in the ECM that affects the airway wall thickness and elasticity.

Chronic obstructive pulmonary disease (COPD) is typified by progressive destruction of the airways and lung parenchyma (Figure 3). This type of lung injury can occur due to genetic predispositions or as a result of environmental exposure to noxious particles or gases [84]. COPD is defined as a progressive lung disease worsened by episodes of acute infective exacerbation. A report published by the Global Burden of Disease Study in 2016 stated that there were over 251 million people with COPD worldwide [85], and COPD is predicted to be the third leading cause of death by 2030 [86]. Cigarette smoke is the most important risk factor for the development of COPD, with a higher prevalence of lung function abnormalities and mortality from COPD than non-smokers. Recent concerns have also been raised over the increasing smoking rates of women and the prevalence rate for COPD in females [87,88]. Conversely however, nearly 15 percent of COPD patients have never smoked, strongly suggesting that other factors including those that are inherited, are linked to the development of COPD. In line with this concept, COPD may arise due to alpha-1 antitrypsin (AAT) deficiency (AATD) [89], a genetic cause of COPD characterized by early onset pulmonary emphysema [90].

COPD is characterised by airway remodelling, which significantly alters the ECM, leading to changes in the airway architecture, resistance and elasticity [91]. ECM remodelling in COPD is brought about by high activity of GAG lyases such as heparinase, hyaluronidase and chondroitinase, resulting in degradation of the GAG components of the ECM proteoglycan [91,92]. This in addition to the activities of NE, results in degradation of elastin and collagen fibres, resulting in significant structural damage of the ECM (Figure 3) [93]. In line with this, circulating serum biomarkers of ECM turnover were significantly associated with COPD disease severity and clinically relevant outcomes [94]. Furthermore, levels of heparin sulphate and CS tightly correlated with upregulation of matrix-metalloprotease (MMP)-2, MMP-9 and MMP-12, which are known to be involved in airway remodelling [95], thus suggesting a potential relationship between GAGs and the remodelling of the respiratory tract in COPD.

As discussed, HA is a major component of the ECM and its level has been found to be altered in patients with COPD [96]. Interestingly, HA has different biological roles depending on its molecular weight. High-molecular-weight HA is known to have immunosuppressive and anti-inflammatory roles contrary to the tissue damaging and pro-inflammatory roles of low-molecular-weight HA [97,98]. Levels of low-molecular-weight HA in BAL of COPD patients have been found to be significantly higher when compared with healthy individuals [99,100]. Furthermore, the rate of HA breakdown correlates with incidence of acute exacerbations of patients with COPD [100].

With regards to the potential therapeutic role of GAGs in COPD, in a murine model of smoke-induced lung disease, treatment with intraperitoneal polysulphated HA reduced BAL IL-1α, IL-2, and TNFα levels, with reduced BAL inflammation and lung permeability recorded [101]. Indeed, investigations into the therapeutic anti-inflammatory properties of high-molecular-weight HA in COPD, included an in vitro study demonstrating that HA had a protective effect on lung elasticity by preventing elastolysis [99]. While no definitive mechanism for this effect was shown, it has been hypothesized that the self-aggregating nature of HA may cause exogenous and endogenous HA to associate and produce higher-MW composites. Nebulized high-molecular-weight HA has been demonstrated to shorten the duration of respiratory failure and to also prevent the requirement of mechanised ventilation during treatment of COPD exacerbations [102]. Finally, nebulized unfractionated heparin has been tested as a treatment for both severe COPD and asthma, although the results of randomized, double-blind placebo controlled trials do not conclusively indicate a strong efficacy [44]. A 2018 study by Shute et al. suggests that increasing the dosage and duration of inhaled heparin treatment can produce clinically relevant improvement in pulmonary function [103].

There are a number of studies that suggest that serum proteoglycans or GAGs may act as potential biomarkers in the course of airway diseases, thus emphasizing their clinical significance. For example, by mass spectrometry analysis, plasma HS levels were significantly elevated in indirect lung injury, while increased HA levels reached statistical significance in patients with direct lung injury [104]. More relevant to COPD, changes in serum levels of proteoglycans or GAGs may reflect remodelling of the lung associated with disease activity. One such study reported on endocan, a dermatan sulphate proteoglycan that is expressed by endothelial cells in alveolar walls of the lung. Serum endocan levels were significantly higher in COPD patients compared to healthy control subjects, however, when patients were classified according to the Global Initiative for Chronic Obstructive Lung Disease (GOLD) criteria, no relationship between GOLD COPD categories and endocan levels was recorded [105]. Further studies were designed to explore the relationship between serum endocan and acute exacerbations of COPD, with significantly increased serum endocan levels detected during an exacerbation, which negatively correlated with forced vital capacity (FVC), forced expiratory volume in 1 s (FEV1), partial oxygen pressure and oxygen saturation [106]. With regards to GAGs, considerably increased serum levels of HS, yet decreased levels of CS, have been reported during an exacerbation as compared to stable-state COPD [107]. To summarize, serum GAGs may be useful biomarkers in the course of airway diseases and exacerbations of COPD, however, further research of large sample sizes are required to corroborate this theory.

### 4.3. Glycosaminoglycans in Asthma

Asthma is a disease of the airways characterised by bronchial hyper-reactivity, allergic airway inflammation and reversible airway obstruction affecting more than 300 million individuals globally [108,109]. GAGs, particularly HA, have been shown to play a key role in the pathogenesis of asthma [99]. Elevated levels of HA are found in BAL fluid of asthma patients, and are significantly correlated with disease severity [110]. The harmful effects of low-molecular-weight HA in asthma are mediated by binding CD44 and receptor for HA-mediated motility (RHAMM) receptors, CD44 and CD168, which precedes the release of pro-inflammatory cytokines [111]. Liang et al. showed that CD44 expression is decreased on macrophages in patients with asthma, resulting in diminished clearance of HA fragments. This is thought to contribute significantly to chronic inflammation [110]. Low-molecular-weight HA was also shown to have potential involvement in inflammation and airway remodelling via upregulation of transformative growth factor-β (TGF-β) and activation of eosinophils [100]. Moreover, studies into the protective effects of high-molecular-weight HA in asthma are scarce. High-molecular-weight HA is known to induce regulatory T-cell activity, thereby supporting anti-inflammatory effects [99]. One study of 14 patients showed that aerosolised high-molecular-weight HA (400–4000 kDa) resulted in decreased exercise-induced bronchial hyper-reactivity [112]. Further studies have shown benefits of HS as a potential therapy for asthma. Ghonim and colleagues demonstrated a favourable effect in a murine model of asthma, reducing allergic-asthmatic inflammation. The potential mechanism proposed for this effect was downregulation of the IL-4/JAK1/STAT6 pathway [113].

### 4.4. Glycosaminoglycans in COVID-19

Emerging from Wuhan, China in late 2019, SARS-CoV-2 quickly became an enormous threat to public health worldwide. Coronavirus disease 2019 (COVID-19), a disease caused by the SARS-CoV-2 virus is highly contagious and can have wide and varying symptoms, most commonly fever, cough, dyspnoea, headache, and/or anosmia [114]. Roughly 5% of patients who contract COVID-19 will progress to acute respiratory distress syndrome (ARDS), characterised by breathing difficulty resulting in hypoxia, the leading cause of mortality in COVID-19 [114,115]. Patients with comorbidities such as diabetes, asthma and obesity are at particular risk of this progression to ARDS [116].

**Figure 4:**
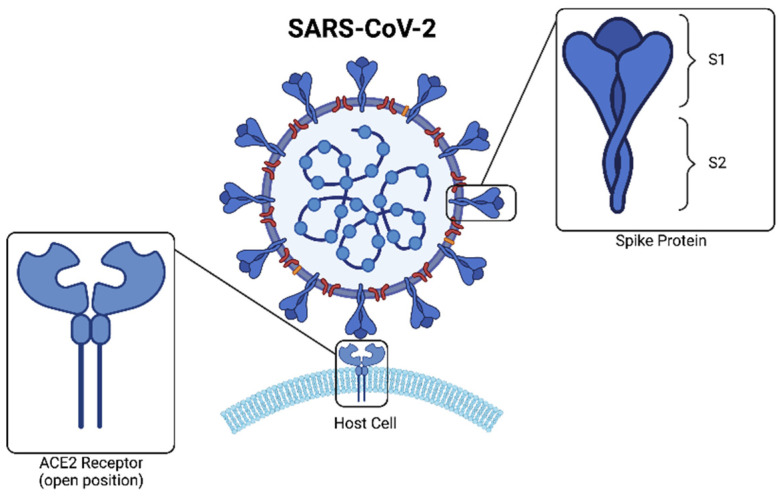
Binding of SARS-CoV-2 to ACE2 receptor: One of the discovered entry routes of the SARS-CoV-2 virus into the host cell is through binding of its spike protein S1 with the ACE2 receptor.

COVID is most commonly transmitted via aerosol droplets (proliferated by coughing, sneezing, and talking) and enters the upper respiratory tract. The surface of SARS-CoV-2 is coated with many spike (S) proteins, which mediate its entry into host cells [117] (Figure 4). SARS-CoV-2 enters host alveolar cells via endocytosis using the angiotensin-converting-enzyme (ACE2), to which the S-protein binds through its receptor binding domain (RBD) located in the S1 subunit [116,117]. Upon entry to the cell, SARS-CoV-2 provokes a host immune response, the severity of which correlates with severity of disease course [118]. It has been shown in several clinical studies that IL-6 is a key driver of disease progression in COVID-19, mediating many of the symptoms of a ‘cytokine storm’, in which an amplified immune response can result in multi-organ dysfunction [119,120,121]. Moreover, a protease–anti-protease imbalance in the airways of patients with COVID-19 has been described [122], a strong target for anti-protease therapy.

**Figure 5:**
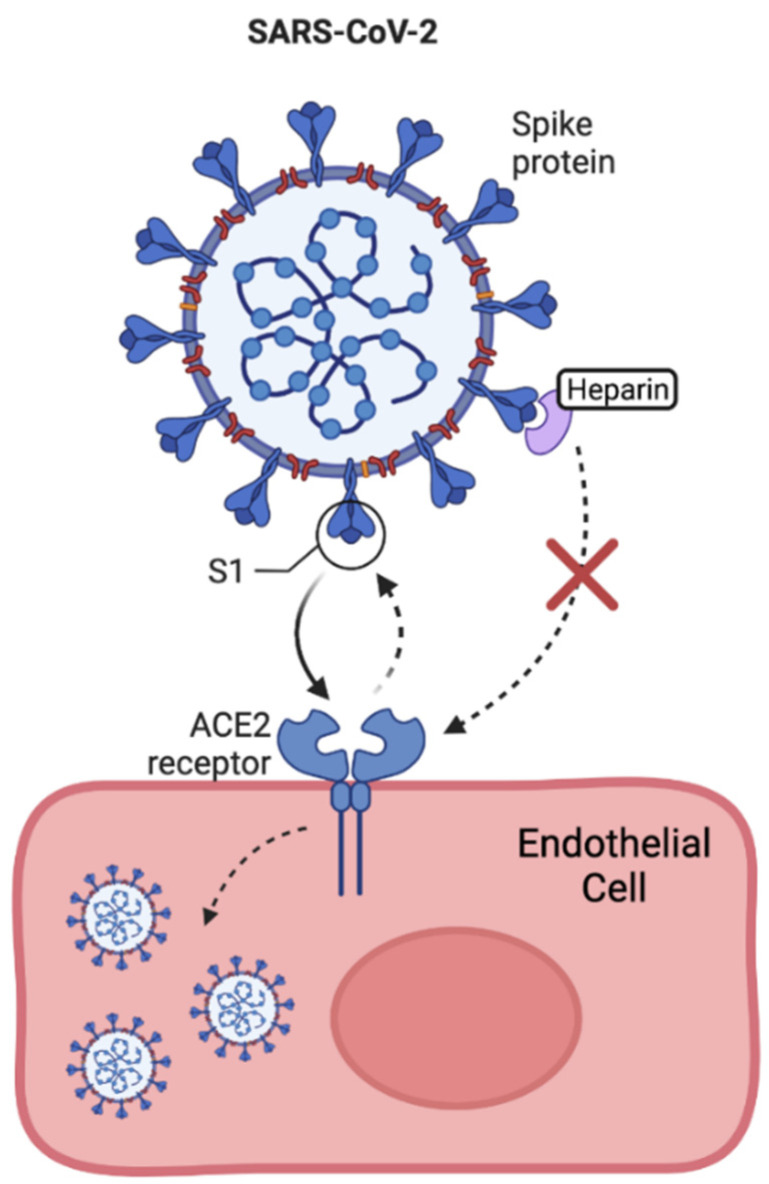
Prophylactic role of heparin in COVID-19 pathophysiology: Binding of low-molecular-weight heparin to the S-protein prevents binding of the S-protein to ACE2 preventing SARS-CoV-2 entry into the host cell and subsequent infection.

It has been demonstrated that SARS-CoV-2 has a putative binding site on its spike protein for GAGs and is able to bind GAGs as co-receptors (Figure 5) [123]. HS proteoglycans are necessary co-factors required for SARS-CoV-2 infection, based on the finding that removal of cell surface HS with heparin lyases dramatically disrupts the binding of the S-protein of SAR-CoV-2 to cell surfaces, thus impairing SARS-CoV-2 infection [124]. This has led to investigations into the therapeutic role of GAGs in COVID-19, based on the ability of GAGs to modulate the activity of a host of proteins and downstream molecular pathways [116].

Clinical reports have shown that endothelial dysfunction is associated with severe COVID-19 cases. The endothelial glycocalyx is a carbohydrate-rich layer lining the vascular endothelium and is enriched with proteoglycans and glycoproteins as well as associating GAGs including HS, HA and CS. In COVID-19, one of the reported contributing factors to vascular injury due to degradation of the glycocalyx is degradation of HA. Circulating hyaluronidase and HA fragments are key signatures of glycocalyx injury and this has been reported to associate strongly with the sequential organ failure assessment scores as well as increased inflammatory cytokine release in patients with COVID-19 [125]. Plasma levels of HS, HA, IL-1β and IL-6 in addition to increased lipid peroxidation have been observed in COVID-19 patients [126]. The release of these inflammatory cytokines and shedding of glycocalyx components are important factors associated with disease exacerbation not only in COVID-19 patients but in other diseases such as cardiovascular diseases and diabetes [127,128].

#### Glycosaminoglycans and the Potential Management of COVID

There has been a growing interest in the use of HS as a potential COVID-19 therapeutic because many viruses including some coronaviruses utilise HS as a coreceptor for attachment to host cell [129]. The interaction between S-protein and cellular HS has been proposed as a potential therapeutic target, with HS being identified as a vital co-factor in SARS-CoV-2 infection. A report by Clausen and colleagues presents electron-microscopic evidence that HS acts as a coreceptor for the docking of SARS-CoV-2’s S-protein to ACE2 [124]. The proposed mechanism for this effect is that HS induces a structural change to the helix and beta-sheets of the S-protein, resulting in the RBD adopting an open conformation that is more receptive to ACE2 [118]. Several in vitro studies have since shown evidence supporting this finding, with a 44–80% reduction in SARS-CoV-2 infection reported on cell lines treated with HS [130] (Figure 5). A further study supporting this concept demonstrated the high-affinity binding of SARS-CoV-2 S-protein to immobilized HS [131]. Interestingly, the presence of low-molecular-weight heparin resulted in a dose-dependent reduction in the binding between S-protein and surface-bound HS, suggesting the potential for the use of low-molecular-weight heparin as a prophylactic for COVID-19 infection. In a further study, the use of unfractionated heparin was explored as a possible safe and effective prophylactic to COVID-19 transmission, applied intranasally [132].

Devising a novel antiviral therapy based on the interaction between HS and S-protein would involve the design of an exogenously competitive HS mimetic in order to modulate the inflammatory response [118]. The two key areas of interest regarding HS as a potential COVID-19 therapeutic have been its anticoagulant and anti-inflammatory properties. Venous thromboembolism is a serious complication of SARS-CoV-2 infection. COVID-19 patients will commonly exhibit hyper-coagulopathy, which can develop into pulmonary thrombosis [133]. In an observational study involving 2574 patients, an Italian study found that heparin treatment was associated with reduced mortality and improved convalescence in severely ill patients with hyper-coagulation [134]. Furthermore, studies have shown that hospitalized COVID-19 patients treated with low-molecular-weight heparin or ultra-low-molecular-weight heparin had improved mortality [135,136]. Highly sulphated HS- and CS-based compounds have also been developed for testing in COVID-19 treatment to block HS proteoglycan interaction with SARS-CoV-2 [137]. The compounds were found to inhibit SARS-CoV-2 growth in cells infected with the virus especially if the compounds were added during adsorption phase. The antiviral mechanism was however found to involve inhibition of viral replication but not binding of the virus to host cell.

Low-molecular-weight heparin also exhibits anti-inflammatory effects that has been explored in different diseases and thus hypothesised to be beneficial in COVID-19 treatment [138,139]. High levels of circulating cytokines are linked to risk of thrombosis and may explain the occurrence of thrombosis and multi-organ failure observed in severe COVID-19 patients [140]. From clinical evidence, IL-6 is the more frequently reported cytokine elevated in COVID-19, which mediates acute inflammatory responses in the disease [141,142]. Low-molecular-weight heparin targeting of IL-6 could thus be used to mitigate the effects of a cytokine storm by reducing the levels of IL-6 preventing disease exacerbation [119]. COVID-19 patients in hospitalisation treated with low-molecular-weight heparin were found to have reduced levels of IL-6 with a simultaneous increase in lymphocytes. This reduction in IL-6 by low-molecular-weight heparin was suggested to have been due to inhibition of NF-κβ, a transcription factor that regulates IL-6 gene expression [119].

### 4.5. Glycosaminoglycans and Non-Infectious Lung Injury

GAGs can play an active role in non-infectious lung injury. Normally HA is a large, biologically inert molecule that provides structure. However, in lung injury or inflammation, it can be found as small-molecular-weight fragments due to changes in synthesis by HA synthases (HAS)-1, HAS-2, or degradation by hyaluronidase (HYAL-1). HAS-1 and -2 synthesise high-molecular-weight HA fragments, whereas HYAL-1 fragments HA [143,144,145]. HA fragments have been shown to act via TLR2-MyD88 or TLR4-MyD88 binding to induce inflammatory chemokines, such as MIP-1α, MIP 1β, KC, RANTES, MCP-1, IP-1, IL-12, TNFα and IL-8 [146,147,148,149]. HA fragments can also markedly induce IL-8 mRNA expression from alveolar macrophages, and both MCP-1 and IL-8 in peritoneal mesothelial cells [150,151]. CD44, a type 1 transmembrane glycoprotein that is expressed on hematopoietic cells is a known receptor of HA [152]. CD44 is necessary for clearing HA fragments that are produced after lung injury, and consequentially, dysfunctional CD44 results in sustained inflammation. CD44-deficient mice demonstrate impaired clearance of apoptotic neutrophils, accumulation of HA fragments and impaired activation of transforming growth factor-beta1, all partially reversed on reconstitution with CD44+ cells [153]. Moreover, improper use of mechanical ventilation has been shown to cause activation of MMPs, that can cleave proteoglycan within the endothelial surface layer [154].

## 5. Conclusions

Within the lung, GAGs play a crucial role in regulating ECM structure, function and the inflammatory response. However, there is increasing evidence of dysregulated GAG expression in the pathophysiology of various lung diseases and continued research investigating the role of GAGs in airway inflammation is required. In this regard, the interaction of inflammatory molecules with specific GAG motifs provides a tool to select specific cytokines and chemokines at the site of inflammation. Moreover, targeted interference with chemokine/cytokine–GAG binding could present a practical therapeutic strategy and an alternative approach to the immunologic treatment with receptor antagonists. Such an approach has been explored for IFN-γ [155], RANTES [156] and IL-8 [157]. Without question, there is an established role for GAGs in CF, as well as COPD, asthma, and COVID-19, both in their pathogenesis and therapeutics. While in vivo studies of the role of GAGs as a therapeutic against SARS-CoV-2 infection are sparse, in vitro studies in combination with the early clinical evidence show encouraging signs of HS being a potential therapeutic option for COVID-19. As more studies emerge, we can expect a deeper insight into the interaction between GAGs and SARS-CoV-2, which may yield new therapeutic targets. However, advantages or possible risks associated with such treatments and their eventual evaluation as possible therapeutics in chronic and inflammatory pulmonary disease have yet to be assessed. We eagerly await further advances in our understanding of the roles of GAGs in airway diseases, that may translate into future novel therapeutics.

## Data Availability

Not applicable.
